# Insulin secretion from beta cells in intact mouse islets is targeted towards the vasculature

**DOI:** 10.1007/s00125-014-3252-6

**Published:** 2014-05-05

**Authors:** Jiun T. Low, Michael Zavortink, Justin M. Mitchell, Wan J. Gan, Oanh Hoang Do, Christof J. Schwiening, Herbert Y. Gaisano, Peter Thorn

**Affiliations:** 1 School of Biomedical Sciences, University of Queensland, St Lucia, QLD 4072 Australia; 2 Department of Physiology, Development and Neuroscience, University of Cambridge, Cambridge, UK; 3 Department of Medicine, University of Toronto, Toronto, Ontario Canada

**Keywords:** Beta cell, Exocytosis, Insulin, Islets, Secretion, Synapse, Vasculature

## Abstract

**Aims/hypothesis:**

We set out to test the hypothesis that insulin secretion from beta cells is targeted towards the vasculature.

**Methods:**

The spatial location of granule fusion was identified by live-cell two-photon imaging of mouse pancreatic beta cells within intact islets, using sulforhodamine B labelling. Three-dimensional (3D) immunofluorescence of pancreatic slices was used to identify the location of proteins associated with neuronal synapses.

**Results:**

We demonstrated an asymmetric, non-random, distribution of sites of insulin granule fusion in response to glucose and focal targeting of insulin granule secretion to the beta cell membrane facing the vasculature. 3D immunofluorescence of islets showed that structural proteins, such as liprin, piccolo and Rab2-interacting molecule, normally associated with neuronal presynaptic targeting, were present in beta cells and enriched at the vascular face. In contrast, we found that syntaxin 1A and synaptosomal-associated protein 25 kDa (SNAP25) were relatively evenly distributed across the beta cells.

**Conclusions/interpretation:**

Our results show that beta cells in situ, within intact islets, are polarised and target insulin secretion. This evidence for an ‘endocrine synapse’ has wide implications for our understanding of stimulus–secretion coupling in healthy islets and in disease.

**Electronic supplementary material:**

The online version of this article (doi:10.1007/s00125-014-3252-6) contains peer-reviewed but unedited supplementary material, which is available to authorised users.

## Introduction

Whether pancreatic beta cells target insulin secretion towards the vasculature is controversial. An early experiment used chronic stimulation to reduce the numbers of insulin granules; the remaining granules were enriched in one region of the cell, suggesting polarisation of either granule trafficking or secretion [1]. Functional support for targeted secretion was obtained from isolated beta cells, which showed polarised calcium responses [2], and, using microelectrode-detection, polarised insulin secretion [3]. However, another study, again in single cells, showed granule exocytosis randomly across the cell membrane [4]. These functional experiments used single cultured cells, which does affect secretory control [5] and removes crucial cell-to-cell contacts found in the islet. One study, in which islets were imaged, suggested that insulin secretion occurs away from the vasculature [6]. But this study was limited to single-plane imaging and did not determine the complex three-dimensional (3D) relationships of the beta cells to the vasculature [1]. Resolution of this issue requires live-cell imaging within intact islets and 3D imaging, something not yet achieved [7].

If insulin secretion was targeted it would require localisation of proteins to direct the trafficking and fusion of secretory granules. In support of this concept, proteins that organise the targeting of presynaptic neurotransmitter release have been found in beta cells [8–12]. However, only one study, on the scaffold protein ELKS [13], immunolocalised the proteins in islets and showed that ELKS appears to be enriched at the vascular face [12].

Why could targeted insulin secretion be important? By analogy with another endocrine cell, the chromaffin cell, targeted secretion could be profoundly important in understanding stimulus–secretion coupling. Chromaffin cells in situ show exocytic responses to single action potentials [14] but cultured chromaffin cells need much higher levels of stimulation to elicit secretion [15]. This suggests that in situ the sites of calcium entry are close to sites of exocytosis; an arrangement that is lost in isolated cells. The in vitro data suggest that the cells integrate inputs whereas the in situ data prove the capacity to respond to single inputs. With reference to beta cells the focal targeting of secretion could have similar consequences. While it appears that some targeting of secretion may be maintained in beta cells in culture [16], studies of models of disease, such as glucose [17] and lipid toxicity [18], suggest that there is separation of the calcium entry channels away from sites of exocytosis. Proof of targeted secretion in beta cells could therefore have widespread implications for normal and diseased beta cell behaviour.

Here, we show that in intact islets, insulin granule fusion is non-randomly distributed across the beta cell membrane and is targeted towards the vasculature. We further demonstrate that proteins associated with the cytomatrix of the active zone in neuronal synapses are found in beta cells and are enriched at the vascular face.

We conclude that beta cells possess an ‘endocrine synapse’ that targets secretion of insulin towards the vasculature.

## Methods

### Experimental solution

Experiments were performed in an extracellular solution (in mmol/l: 140 NaCl, 5 KCl, 1 MgCl_2_, 2.5 CaCl_2_, 5 NaHCO_3_, 5 HEPES, glucose) adjusted to pH 7.4 with NaOH.

### Islet preparation

CD1 mice (local colony) were humanely killed according to local, University of Queensland, animal ethics procedures (approved by the University of Queensland, Anatomical Biosciences Ethics Committee).

### Islet slices

Sectioning of unfixed pancreatic tissue was performed as previously described [19]. See electronic supplementary material (ESM) Methods for details.

### Antibodies

A range of primary antibodies were used. See ESM Methods for details.

### Western analysis

Standard western blot methods were employed. See ESM Methods for details.

### Cultured islets

Isolated mouse pancreatic tissue was prepared by a collagenase (type IV) (Worthington, Lakewood, NJ, USA) digestion method in Hanks’ buffer (Sigma-Aldrich, Castle Hill, NSW, Australia) adjusted to pH 7.4 with NaOH. Isolated islets were maintained (37°C, 95/5% air/CO_2_) in RPMI-1640 culture medium (Sigma-Aldrich) containing 10.7 mmol/l glucose, supplemented with 10% FBS (Gibco, Life Technologies, Mulgrave, VIC, Australia), 100 U/ml penicillin and 0.1 mg/ml streptomycin (Invitrogen, Life Technologies, Mulgrave, VIC, Australia).

### Islet imaging

Isolated islets of Langerhans were cultured for 2–3 days and, before imaging, were bathed in an extracellular solution containing 3 mmol/l glucose for 30 min (37°C, 95/5% air/CO_2_). Two-photon imaging was performed at 34°C with exocytic events recorded as the entry of extracellular dye into each fused granule.

### Two-photon imaging

A custom-made microscope was employed. See ESM Methods for details.

### Statistical analyses

Data are presented as mean ± SEM. Statistical analysis was performed using Microsoft Excel 2010 (Microsoft, Redmond, WA, USA) and GraphPad Prism (ver 6, GraphPad Software Inc, La Jolla, CA, USA). Data were subjected to a Student’s *t* test. Significance is indicated as *p < 0.05, **p < 0.01 or ***p < 0.001. Islets from at least three mice were used in each experiment.

## Results

### Live-cell imaging of single insulin granule fusion in islets

**Fig. 1:**
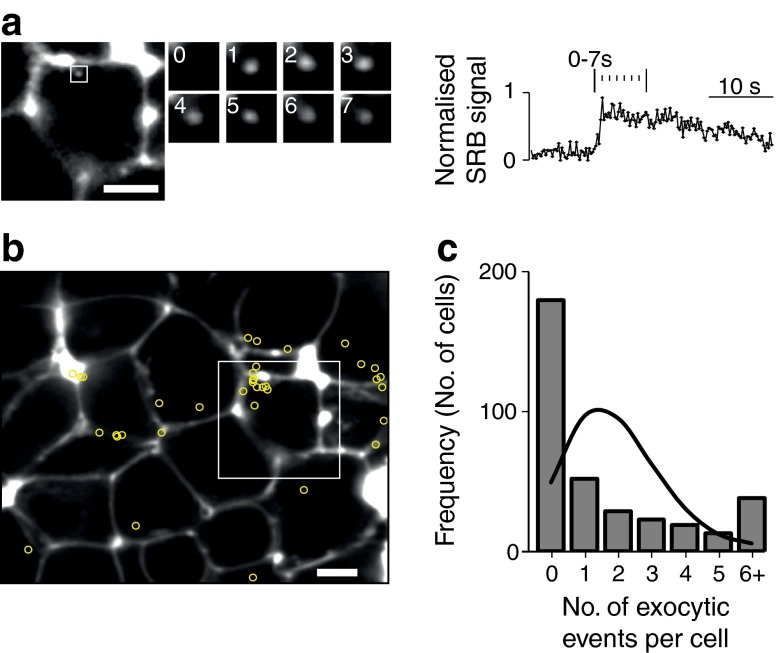
Live-cell imaging of insulin granule fusion. (**a**) The images show a single cell within an isolated islet. The extracellular fluorescent dye SRB outlines the cell and when insulin granule fusion occurs this dye enters the granule, shown as time-sequence images taken at 1 s intervals. The average fluorescence changes within a region of interest placed over the fusion granule show a characteristic rapid increase in fluorescence after fusion and then typically show a slow decay; scale bars 5 μm. (**b**) Beta cells, within the two-photon cross-section show a non-random distribution of secretory responses to high glucose. Insulin granule exocytosis was induced in islets by exposure to high glucose (8–15 mmol/l) for 20 min. The location of each exocytic event is marked by a yellow circle and shows an uneven distribution across the cells; some cells show many events and some have none; scale bar, 5 μm. The frequency histogram (**c**) shows that the majority of the cells have no exocytic events but that some cells have a high number of exocytic events when compared with the Poisson distribution (line on histogram)

We used isolated, cultured, mouse pancreatic islets bathed in extracellular solution supplemented with sulforhodamine B (SRB) and imaged with two-photon microscopy. The dye outlines each cell and enters each fusing granule following stimulation with glucose. We observed these fusion events as the sudden appearance of bright spots of fluorescence (Fig. 1a). All live-cell experiments were performed on cultured, isolated islets.

This method has been used previously [6, 20] and, in principle, provides a non-specific label for vesicular dye uptake into cells. However, our published evidence [21] indicates that under our conditions we are likely to be almost exclusively recording insulin granule fusion in beta cells. For example, we record three cell layers into the islet where almost all the cells in a rodent islet are beta cells, the size of the vesicle events we observe is consistent with insulin granule size, the numbers and time course of fusion events are consistent with the amount of insulin secretion, the size of the cells are consistent with beta cells and we record from cells that post-experiment immunostain for insulin [21].

### Glucose-induced exocytosis of insulin granules is non-uniform

Increasing extracellular glucose concentration from 3 to 15 mmol/l induced many insulin granule fusion events. Each fusion event taking place over a 20 min period was identified (using the fluorescence signatures as in Fig. 1a) and its spatial position was located (Fig. 1b). Our images show that the number of exocytic events is unevenly distributed from cell to cell. Simple geometry (assuming spherical cells) shows that the area of cell membrane, sampled across the two-photon imaging slice (∼1–2 μm deep), is the same wherever in the cell the cross section is made [21]. This means the large cell-to-cell differences in the number of fusion events cannot be explained by differences in the sampled cell membrane areas.

A *χ* ^2^ analysis showed that the observed distribution of exocytic events in each cell was significantly different (*p* < 0.01) from a random, Poisson distribution of events (Fig. 1c; 18 islets, six mice, 360 cells responses to 8 and 15 mmol/l glucose).

**Fig. 2:**
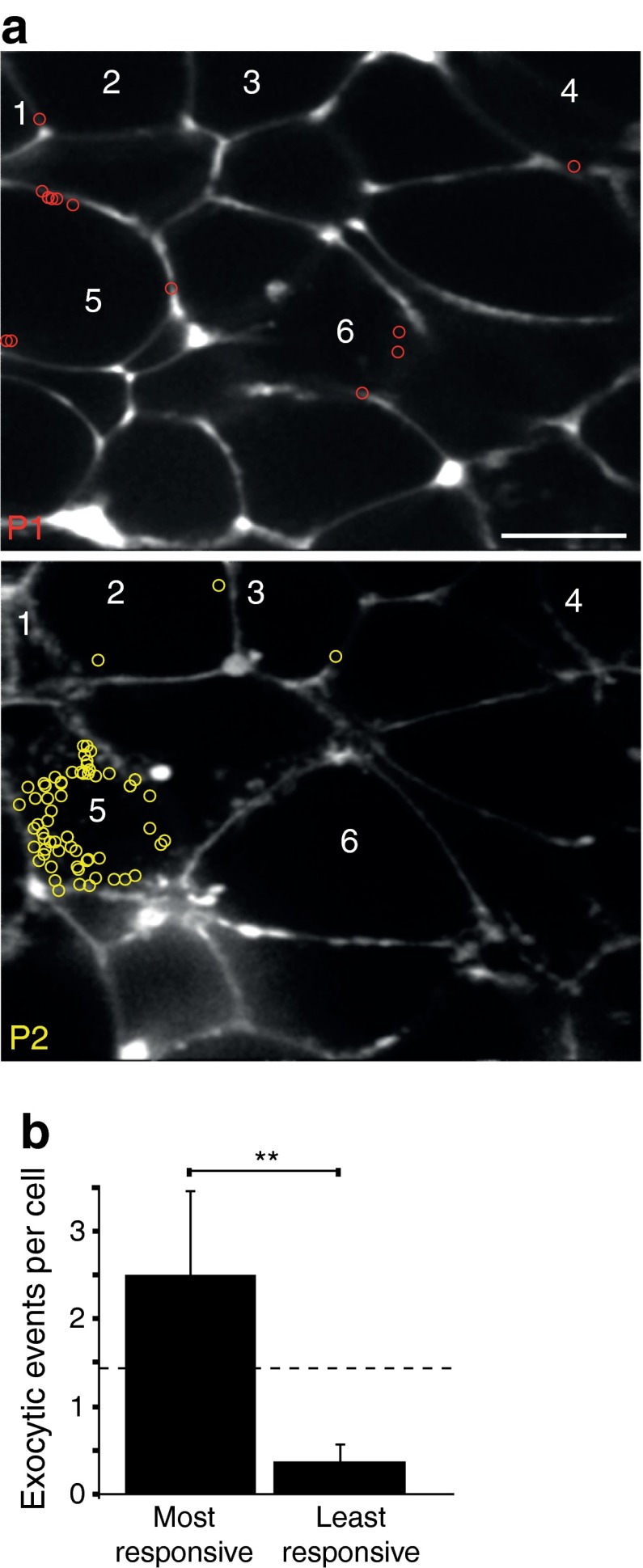
Two-photon imaging at two planes shows an uneven distribution of exocytic events across single cells. Islets were stimulated for 20 min with 15 mmol/l glucose and the location of each exocytic event was marked with a coloured circle (**a**). Plane 1 (P1) was recorded for the first 10 min and then the focus was changed (by 5.5 μm) and plane 2 (P2) was recorded for the next 10 min. Some cells were observed only in one plane; those observed in both planes are numbered 1–6 and all cells, particularly cell 5 in this record, show asymmetric numbers of fusion events in the different planes. (**b**) Histogram of the average number of exocytic events where, for each cell, the most responsive planes and least responsive planes are grouped together. The dotted line shows the number of exocytic event averaged across both planes, which would be the expected observation if the events were evenly distributed. (**b**). Data from four islets and 65 cells. Scale bar, 10 μm

Given that apparently ∼50% of cells did not respond to glucose, we tested whether exocytosis might be occurring in regions of the cell outside the two-photon slice. To do this we sampled at two optical planes, separated in depth by 5.5 μm, during a response to 15 mmol/l glucose (Fig. 2a, b; *n* = 4 islets). In the cells observed in both planes the number of apparently non-responding cells decreased from 54% observed with one optical plane to 44% when observed with two planes (*n* = 37 cells). This suggests that cells are responding but have an uneven distribution of exocytosis around the cell such that a single optical plane may lie outside the region where responses occur.

To further test for an uneven distribution of exocytosis we determined the number of fusion events in one two-photon plane to the number, seen in the same cell, in the other plane (Fig. 2b) and then expressed these numbers as a ratio. An even distribution would predict a 1:1 ratio; in contrast, we measured a 1:6.6 ratio (*n* = 65 cells, 199 exocytic events, paired Student’s *t* test *p* < 0.01). However, exocytic activity changes over time [21] and differences could be due to capturing a burst of activity in one plane and not in the other. Against this idea, by recording each plane for >6 min, which is longer than a single burst [21], our data will temporally average any burst responses. Furthermore, the actual data does not show a systematic bias; 33 cells had the same response, 19 cells had more events in the second plane and 13 had more events in the first plane. We conclude that exocytosis is unevenly distributed around a beta cell.

### Insulin secretion is targeted to the vasculature

**Fig. 3:**
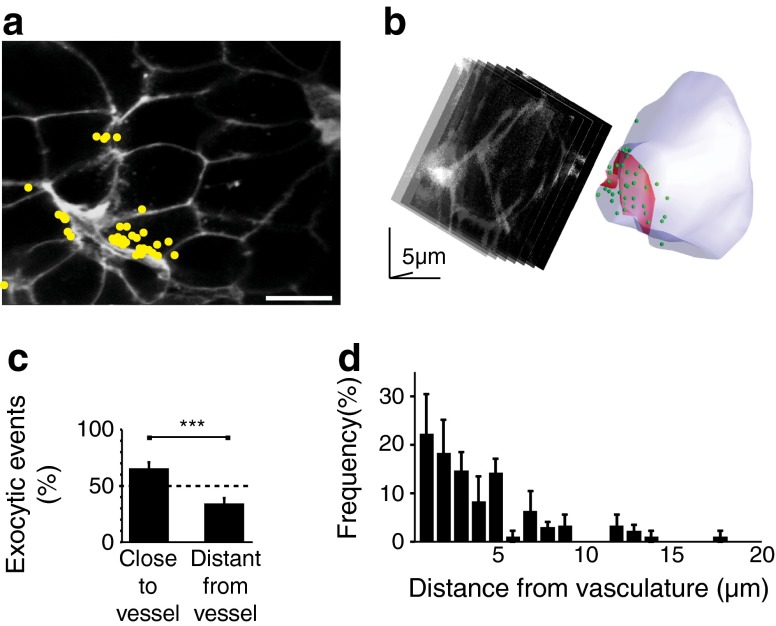
Two-photon live-cell imaging reveals targeting of insulin granule exocytosis to the vascular pole of beta cells. (**a**, **c**) Two-photon imaging shows clustering of sites of exocytosis (recorded over 20 min, in response to 15 mmol/l glucose) towards the fissures that run through the islets (stained brightly with extracellular fluorescent dye) (**a**) and analysed as a histogram (**c**). The histogram shows the proportion of exocytic events in the half of the cell close to the blood vessel compared to the half of the cell that is distant from the blood vessel. The dotted line on the histogram is the expected 50:50 proportion, if exocytosis was evenly spread across the cell. Scale bar, 10 μm. (**b**) Two-photon image capture while sequentially stepping image planes through a single cell enabled identification of the exocytosis of individual insulin granules (shown in the reconstruction as green dots) in space, in response to high-K^+^ stimulation. These exocytic fusion events cluster at the cell membrane in a region adjoining the vasculature (stained with isolectin B4 and shown in red on the reconstruction). (**d**) A histogram of the frequency of exocytic fusion events in relation to the distance from the vasculature shows an asymmetric response with a bias towards the vascular face of the beta cells

Tomographical imaging of islets shows fissures running through the islet, presumed to be vascular in origin [6]. The vast majority of cells have discrete points of contact with the vasculature (only 2% have no apparent contact) but in any two-photon plane points of contacts were observed in only a subset of cells (Fig. 3a). To examine the relationship between the sites of insulin granule exocytosis and the vasculature we therefore analysed only those cells contacting vascular structures (Fig. 3a). Dividing these cells into two regions of equal area, one adjacent to the vasculature and one away, showed that there were significantly more exocytic events in the area closer to the vasculature (Fig. 3c, Student’s *t* test, *p* < 0.001, 13 islets, five mice, 39 cells).

To determine whether these fissures are vascular we counter-stained live cells with isolectin B4, which reacts with α-d-galactopyranosyl groups [22] in the basement membrane [23, 24]. This stained the fissures (ESM Fig. 1) and, using immunostaining, overlays with laminin (ESM Fig. 1).

The above analysis supports the idea of targeted secretion but further evidence requires 3D real-time acquisition of exocytic events over the entire volume of single cells. This is technically challenging, given the short lifetime and small size of each exocytic event. To facilitate our experiments we employed high-K^+^ stimulation, in isolated islets, to rapidly induce a large number of exocytic responses and captured images over 9 min of continual cycling of sequential Z planes (2 μm steps, seven steps per stack, 3 s per stack) through a single cell. Isolectin B4 stained the basement membrane and identified the cell’s orientation with respect to the vasculature. The time and location of each individual exocytic event was identified and plotted out in a 3D representation of the cell (Fig. 3b).

Finally, for each cell (*n* = 4 islets, four cells), we trigonometrically measured the distance between each insulin granule fusion site and the location of the nearest point on the vasculature. The histogram shown in Fig. 3d demonstrates a strong bias for fusion events to be targeted to the vascular pole of the beta cells.

### Polarisation of beta cells towards the vasculature

We conclude that most pancreatic beta cells respond to glucose. The apparent heterogeneity in cellular responses in (e.g. Fig. 1) is due to an uneven spatial spread of granule fusion events across the cell, with preferential targeting towards the vasculature. Drawing on work in neurons, we next tested for the mechanisms that might support this targeting. Previous work has used single sections to immunolocalise proteins in the stimulus–secretion cascade [12]. However, to understand the 3D relationship within the islet we have employed a method of ‘thick’ sectioning (∼100 μm) of islet tissue and serial confocal imaging [25]. All immunostaining work was conducted on these islet slices.

**Fig. 4:**
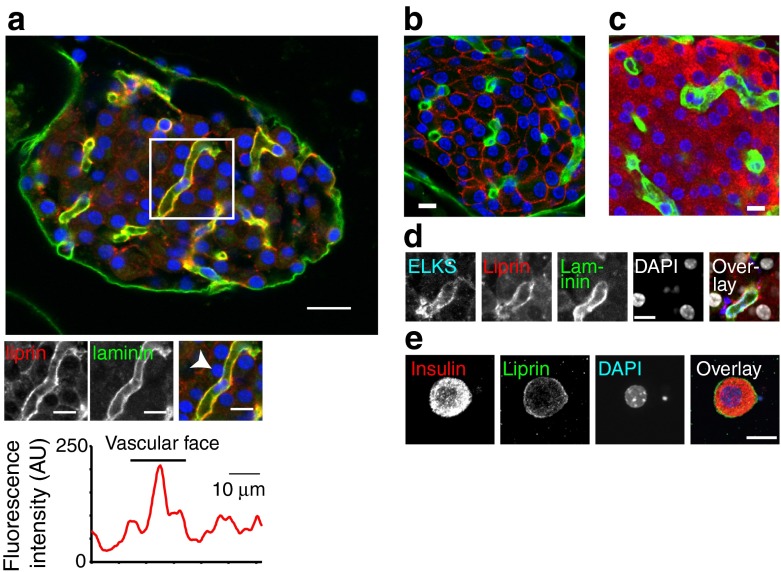
Presynaptic scaffold protein liprin is present in beta cells and is enriched at the vascular face. (**a**) Immunofluorescence image of islets shows laminin (green) as a marker of the basement membrane from the vascular endothelial cells and shown as low power, large images of a mouse whole islet (scale bar, 20 μm) and enlarged images of the regions bordered by the boxes (scale bar, 10 μm). Immunofluorescence of liprin (red) shows enrichment along the vasculature using a linescan around the cell (indicated by arrow) perimeter. (**b**, **c**) GLUT2 (red) and laminin (green) (**b**) and insulin (red) and laminin (green) (**c**) immunostaining show that GLUT2 and insulin are on the membrane of all cells in the islet core proving they are beta cells. (**d**) An isolated beta cell, with insulin immunostaining (red) and liprin (green) at the cell membrane. (**e**) Quadruple immunostaining shows that ELKS and liprin are enriched along the laminin-stained vasculature. Scale bar, 10 μm

3D reconstruction shows the blood vessels (immunostained with laminin) coursing between the cells (Fig. 4). Counter immunolocalisation of the presynaptic scaffold protein, liprin 1α, showed preferential enrichment at the vascular face of beta cells (Fig. 4a and ESM Video 1). The core of a mouse islet is composed almost entirely of beta cells as shown by GLUT2 (only present in beta cells [26]) (Fig. 4b) and insulin (Fig. 4c) immunostaining. Control experiments confirmed that liprin is found in isolated beta cells (Fig. 4e). Quadruple immunostaining showed that ELKS, as previously shown [12], and liprin are enriched along the vasculature (Fig. 4d). Western blots showed that the primary antibodies identified proteins of the correct molecular mass (ESM Fig. 2).

**Fig. 5:**
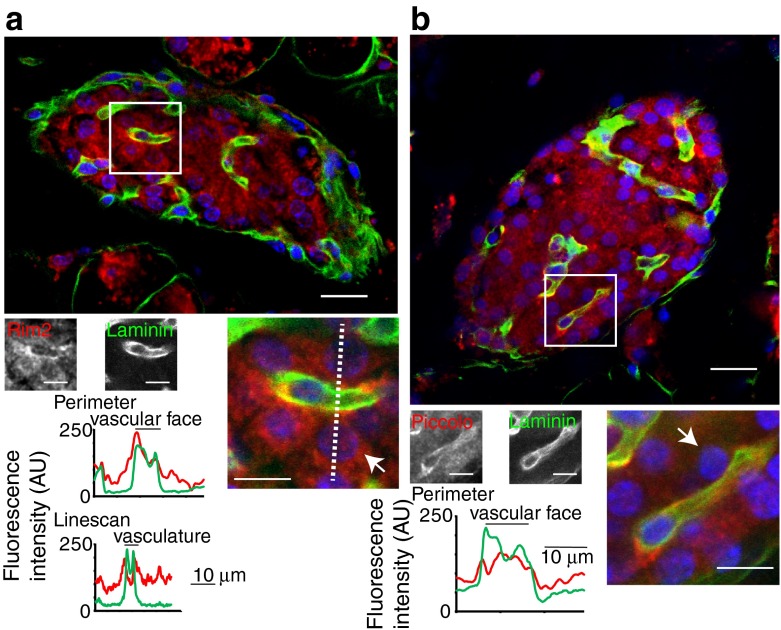
The presynaptic scaffold proteins Rim2 (**a**) and piccolo (**b**) are specifically enriched at the beta cell membrane that borders the vasculature. This enrichment is quantified in the histograms of the average fluorescence intensities either along a line drawn around the perimeter of the cells (indicated by arrows) or along a linescan (shown by the dotted line). Scale bars, 10 μm

A similar staining pattern was seen for Rab2-interacting molecule (RIM2) (Fig. 5a) and piccolo (Fig. 5b), although both are also apparently also diffusely located in the cell cytosol. Further quantification of the distribution of these proteins is shown in the histograms shown in Fig. 5, where the average fluorescence along a line drawn around the perimeter of the beta cells, or as a linescan, shows that enrichment of these proteins is spatially coincident with the staining for laminin (Fig. 5 and ESM Fig. 3). Since these proteins are known to be part of the presynaptic machinery in neurons [13], the polarised expression of these proteins in the islets implies that a similar ‘presynaptic’ complex is present in beta cells.

**Fig. 6:**
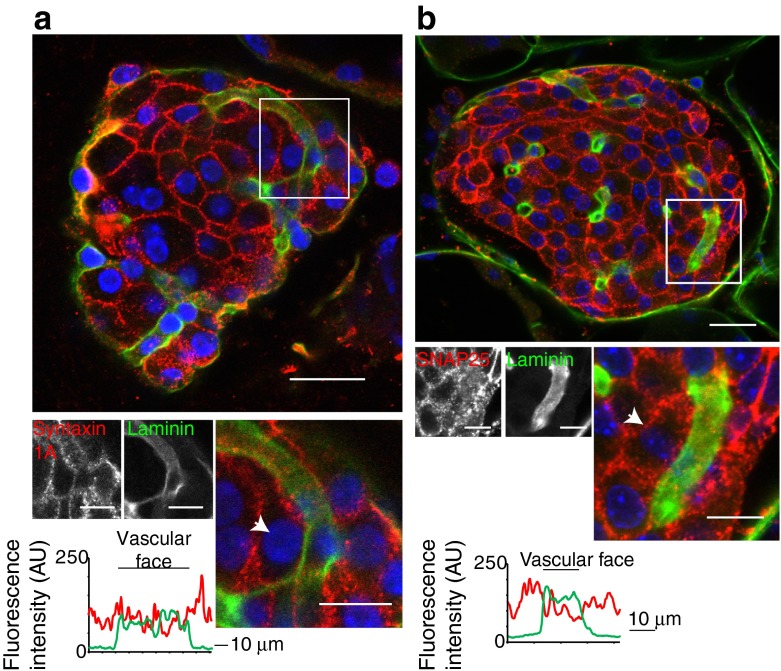
The SNARE proteins syntaxin 1A and SNAP25 are relatively uniformly distributed around the beta cell membrane. (**a**, **b**) Low and high power images of the immunofluorescence for laminin and counter immunofluorescence of syntaxin 1A (**a**) and SNAP25 (**b**). The histograms of the average fluorescence intensity along a line drawn around the perimeter of the cells (indicated by an arrow) show no enrichment at the vascular face of the beta cell. Scale bars, 10 μm

Interestingly, 3D immunostaining of the exocytic soluble *N*-ethylmaleimide-sensitive factor attachment protein receptor (SNARE) proteins, syntaxin 1 (Fig. 6a) and synaptosomal-associated protein 25 kDa (SNAP25) (Fig. 6b), both of which have been previously identified in islets [27, 28], show punctate staining that is subplasmalemmal but is not differentially localised with respect to the vasculature.

## Discussion

We show that in intact islets insulin granule fusion is targeted towards the vascular face of beta cells. At the same vascular face we show enrichment of proteins known to be components of the presynaptic region of neurons. Together these findings provide functional and structural evidence that insulin secretion is dependent on an ‘endocrine synapse’ that targets hormonal outflow to the bloodstream.

### Functional assessment of targeted insulin secretion

Our work is the first functional assessment of the spatial distribution of insulin granule exocytosis in three dimensions within intact islets. Our conclusion of targeting is consistent with electron microscopy results showing that granules were evenly distributed in resting beta cells (ruling out a simple explanation for polarised secretion) but were asymmetrically clustered in chronically stimulated beta cells [1]. More recent experiments measuring signalling or exocytosis in isolated beta cells either support [2, 3] or go against [4] the idea of polarised secretion. However, results from single, cultured cells reflect the culture conditions [5] and are remote from the native environment of the islet.

The work that resembles ours most closely used intact islets and two-photon microscopy, but only took images in one plane [6]. The authors state that ‘most exocytotic events […] occurred in the “abvascular” compartment’ (see their Fig. 2d) (i.e. away from the blood vessels) and this is how their work has been interpreted by others [29]. However, they also state that they ‘detected a polarity of β cells […]: exocytosis occurred preferentially in the vicinity of certain vessels, possibly veins’. These two statements appear contradictory. These experiments were a minor part of this paper and we contest that the use of a single image plane distorts image interpretation. For example, in the most extreme case, an exocytic event could appear to be a beta cell diameter (16 μm [21]) away from a blood vessel in a single plane but, in our 3D experiments, a blood vessel identified immediately above, or below, in the next image plane, would place the exocytic event only 2 μm away (the depth of our Z plane). 3D analysis is therefore essential to map these spatial relationships and reach conclusions about targeting of exocytosis.

We have yet to consistently record exocytosis in tissue slices and therefore performed the functional experiments on cultured islets. It is known that the number of endothelial cells decreases over time in culture [24, 30] and consistent with this we observe a gradual decrease, but not loss, of isolectin B4 staining over days. We conclude that islets remodel in culture but that laminin is still present along the remaining blood vessels and the beta cells adjoining these regions still maintain targeted secretion.

A further point of discussion is our use of high potassium in the 3D measurements. The relationship between high potassium and glucose-induced responses is not clear [8, 31]. We expect that both would be spatially localised and require the same scaffold proteins, although there may be differences in the contributions of specific SNAREs [32]. However, it is possible that there are spatial differences and, furthermore, it is possible that modulators, such as GLP-1, could control different spatial patterns of exocytosis through influences on proteins like RIM2 [8]. Future experiments will be required to investigate these interesting possibilities.

### Molecular mechanisms of the endocrine synapse

There are a few studies identifying proteins in beta cells that are enriched presynaptically in neurons and form a presynaptic stimulus–secretion complex. These include ELKS [12], RIM2 [8], Munc 13-1 [33], SNAP25 [34] and neurexin [11]. However, only ELKS has previously been localised within an islet.

Part of this protein complex is likely to include calcium channels and single-cell work shows calcium entry is polarised [2]. We were unsuccessful in immunolocalising calcium channels but the spatial association of calcium channels is important in secretory control [35] and is a possible factor in disease [18].

Since the differential distribution of SNAREs is the dominant hypothesis to explain polarised targeting of secretion in the pancreatic acinar cells [36, 37], it is interesting that syntaxin 1A and SNAP25 are relatively uniformly distributed around beta cells (Fig. 5). However, the specific localisation of just one other crucial component could dictate the targeting of secretion. Our work points to liprin, RIM2 or piccolo as possible candidates for this essential component. An alternative explanation is that syntaxin isoforms other than 1A are regulators of insulin secretion [32, 38, 39].

### Polarisation of beta cells

Location of these presynaptic proteins to the vascular side of beta cells suggests that the cells are using orientation cues, one of which may be interaction with endothelial cells. During islet development vascular endothelial growth factor A secreted from beta cells is key to endothelial cell location and vascular development [23, 40]. In turn, the basement membrane protein laminin regulates beta cell functions such as insulin gene expression and proliferation [23]. Interestingly, in islet transplant studies, revascularisation of the donor islets is maximal at 14 days which temporally correlates with a return to normoglycaemia [30]. Factors such as hypoxia may be important, but this recovery of functionality would be consistent with the formation of new beta cell–endothelial cell contacts, something directly shown in electron microscopy studies [30].

Extending the analogy with neuronal synapses, we speculate that the beta cell–endothelial cell interactions may use similar recognition mechanisms to a synapse. One candidate is the neurexin–neuroligin pathway in which protein–protein interactions span the presynaptic to postsynaptic cells [41]. Neurexins and neuroligins are found in beta cells [11] and endothelial cells [42] although their in situ distribution has yet to be determined.

### Consequences of secretory targeting for islet function

Our work has implications for the intra-islet effect of insulin. Insulin has feedback actions on beta cells [43]; secretion into the bloodstream makes it unlikely that cells would sense their own secretory output. Instead, beta cells might sense circulating insulin, an argument recently proposed on the basis of computer modelling [44]. In human islets beta cells are scattered throughout the islet (not just in the core as in rodents) [45] but, if vascular targeting of insulin secretion occurs in humans, this would still affect its paracrine/autocrine actions.

Given the importance of spatial control of secretory output, there might be further mechanisms of control that selectively target secretion to the lateral, beta cell-to-beta cell, interface. As well as insulin this could also include the secretion of γ-aminobutyric acid, ATP and zinc, which have known actions within the islet [46–48]. Since disease is associated with changes in beta cell structure [49] and remodelling of the vasculature [50], any mistargeting of secretion could be a component in disease.

### Conclusions

We conclude that our work provides evidence for targeted insulin secretion, which has wide-ranging implications for the control of insulin secretion and for the role of the beta cell within islets. As such its preservation will be important for islet transplant therapy [30] and its establishment important in therapies designed around genetically engineered cells.

## Electronic supplementary material

Below is the link to the electronic supplementary material.

ESM Methods (PDF 53 kb)

ESM Fig. 1 (PDF 1260 kb)

ESM Fig. 2 (PDF 108 kb)

ESM Fig. 3 (PDF 19 kb)

ESM Video 1 A sequence of Z stack images taken through the islet showing immunofluorescence (upper images) of laminin (green) and liprin (red). The lower image shows the liprin staining alone. (MOV 1707 kb)

## Abbreviations

3D: Three-dimensional
RIM2: Rab2-interacting molecule
SNAP25: Synaptosomal-associated protein 25 kDa
SNARE: Soluble *N*-ethylmaleimide-sensitive factor attachment protein receptor
SRB: Sulforhodamine B
