# Supplementary material for: Insulin secretion from beta cells in intact mouse islets is targeted towards the vasculature
Source: Diabetologia. 2014 May 5;57(8):1655–63. doi: 10.1007/s00125-014-3252-6 (PMC4079948; doi:10.1007/s00125-014-3252-6)
Supplement: Supplementary file 1 — (PDF 53 kb) [file 125_2014_3252_MOESM1_ESM.pdf]

Electronic Supplementary Material

Methods

Islet slices Sectioning of unfixed pancreatic tissue was performed as described by Huang et al [1]. Briefly, after cervical dislocation, the pancreas of 10-12 week old CD1 male mice was injected with 1.9% low melt agarose (UltraPure LMP, Invitrogen) in extracellular slice medium (ECSM, 125 mmol/l NaCl, 2.5 mmol/l KCl, 1.25 mmol/l NaH_2PO_4, 26 mmol/l $NaHCO_3$, 2 mmol/l sodium pyruvate, 0.25 mmol/l ascorbic acid, 2mmol/l myo-inositol, 1mmol/l $MgCl_2$, 2mmol/l $CaCl_2$, 6 mmol/l lactic acid and 6 mmol/l glucose at pH 7.4). The common bile duct was clamped at the junction with the duodenum to prevent agarose from entering the small intestine and a 30 gauge needle was used to inject 1-3 milliliter of 42°C agarose through the bile duct to backfill the pancreas. The pancreas was immediately cooled with ice cold ECSM, removed from the mouse, and immersed in ice cold ECSM in a petri dish. Four to 6 mm cubes of this tissue were embedded in 4% low melt agarose in ECSM, immersed in 4°C ECSM and sectioned with a Zeiss (Thermo-Fisher) Hyrax V50 vibrating microtome. Sections (90-100 μm thick) were cut with the instrument set at an amplitude of 0.7 and frequency of 95 and a speed of 4 μm/sec and sections containing uncut islets were stored in ECSM (oxygenated by bubbling with 5% carbogen gas) at 4°C, for no longer than 10 minutes, before fixation. Fixation with 4% paraformaldehyde (Sigma-Aldrich) in ECSM was either for 10 minutes (short PFA) or one hour (long PFA) at 20°C. Slices fixed with methanol were rapidly immersed in -20°C methanol and stored in a freezer for one hour. Methanol-fixed slices were rehydrated in ECSM and then PBS. Slices were stored in either PBS or ECSM at 4°C for up to one week before antibody treatment.

Immunofluorescence was performed as described by Meneghel-Rozzo et al [2]. Sections were incubated in block buffer (3% BSA, 0.3% donkey serum, 0.3% Triton X-100) for a minimum of one hour at room temperature followed by primary antibody incubation at 4° C overnight in block buffer. Typically 4-6 slices were incubated in 0.5 ml block buffer in 1 well of a 6 well dish. Sections were washed in PBS (4 changes over 30 minutes) and secondary antibodies (in block buffer) were added for 4-6 hours at 20° C. After washing in PBS, sections were mounted in Prolong Gold anti-fade reagent (Invitrogen) and imaged on an Olympus Fluoview FV1000 confocal microscope using a UPlanSApo 60X 1.35 N.A. oil objective.

Antibodies Primary antibodies used for this study were

**antibody**	**Company and catalogue #**	**Fixative used**
mouse anti-SNAP25	Synaptic Systems 111 011	PFA
mouse anti-syntaxin 1A	Synaptic Systems 110 111	methanol
rabbit anti-Liprinα1 (PPFIA1)	Proteintech 14175-1-AP	PFA and methanol
rat anti-β1 laminin	Thermo Scientific MAS5-14657	PFA and methanol

rabbit anti-piccolo	Synaptic Systems 142 003	Methanol
rabbit anti-RIM2	Synaptic systems 140 103	Methanol
rabbit anti-PPFIA1	liprin 1α, Proteintech 14175-1-AP	Methanol
rabbit anti-insulin	Genesearch 3014S	PFA
Mouse anti-insulin	Sigma I2018	PFA and methanol
rabbit anti-GLUT2	Merk 400061	PFA
mouse anti-ELKS	Abcam ab50312	PFA

All primary antibodies, except the laminin antibody, were diluted 1/200, the anti-laminin antibody was used at 1/500. Secondary antibodies were highly cross absorbed donkey or goat antibodies (Invitrogen) labelled with Alexa 488, Alexa 546 or Alexa 633. All were used at a 1/200 dilution. Where used, DAPI (Sigma, 100 ng/ml final concentration) was added for the last 2 hours of secondary antibody incubation. nb rabbit anti-insulin was used in islets and mouse anti-insulin used in isolated cells.

Western analysis Brain samples were prepared by lysis of 0.2 gram of frozen CD1 mouse brain in 0.2 milliliter of RIPA buffer (25mmol/l Tris-HCl pH 7.6, 150 mmol/l NaCl, 5 mmol/l EDTA, 1% Trition X-100, 1% sodium deoxycholate, and 0.1% SDS) including a protease inhibitor cocktail (Roche complete 11836153001) and PMSF (1 mmol/l). Tissue was disrupted with a dounce homogenizer, left on ice for 5 minutes, re-homogenized, and centrifuged to remove cellular debris. BCA assays (Biorad) were used to determine protein concentration using BSA as a standard. Because of limited islet tissue, freshly isolated islets were accumulated at -80C, then combined and lysed in RIPA buffer. The volume of the lysate was adjusted with concentrated sample buffer to load approx 170 islets per lane. Brain samples (10 µg total protein) and islets were run on 4-15% mini protean TGX precast gel (Biorad) and transferred to immobilon-FL PVDF membrane (Millipore). Antibody detection was done with Li-Cor Odyssey blocking buffer using standard protocols with IRDdye 680 and 800 coupled secondary antibodies (Li-Cor) and the membrane scanned on a Li-Cor Odyssey Infrared imaging scanner.

Two-photon imaging We used a custom-made, video-rate, two-photon microscope with a 60x oil immersion objective (NA 1.42, Olympus). A sub-resolution bead (100 nm diameter) had a Z spread (full width, half maximum) of 1.85 +/- 0.48 µm. We imaged exocytic events using Sulforhodamine B (SRB, 800 µM/l) as a membrane-impermeant fluorescent extracellular marker excited by femtosecond laser pulses at 950 nm, with fluorescence emission detected at 550-650 nm. 3D images were collected at a frame rate of 6 Hz with Z sections 2 µm apart.

Images (resolution of 10 pixels/µm) were captured using ScanImage software [3] controlling custom hardware and analysed with the Metamorph program (Molecular Devices Corporation,

USA). Exocytic event kinetics were measured from regions of interest (0.78 μm^2, 78 pixels) centred over individual granules.
Traces were rejected if extensive movement was observed. In the 3D analysis we were concerned to reject exocytic events that might be recorded in more than one image slice; ie one single event that would be counted twice. Analysis showed that all exocytic events, except one, appeared to arise from separate regions in each image slice. As an additional criterion, we rejected all events that were less than half the fluorescent amplitude of the brightest 3 events in any slice. These sub-threshold events were a minor component of the overall response <10% and were not systematically located in any particular region of the cell.

The linescan analysis in Fig. 5 was performed using Metamorph with the average fluorescence taken along a 2.07 μm width and a line drawn either around the cell perimeter (identified with a counter immunostain for syntaxin 1a) or with a linescan across the vasculature.

References

[1] Huang YC, Rupnik M, Gaisano HY (2011) Unperturbed islet alpha-cell function examined in mouse pancreas tissue slices. J Physiol (Lond) 589: 395-408

[2] Meneghel-Rozzo T, Rozzo A, Poppi L, Rupnik M (2004) In vivo and in vitro development of mouse pancreatic beta-cells in organotypic slices. Cell Tiss Res 316: 295-3031

[3] Pologruto TA, Sabatini BL, Svoboda S (2003) ScanImage: Flexible software for operating laser scanning microscopes. Biomed Imag Online 2:13
