# Supplementary material for: Insulin secretion from beta cells in intact mouse islets is targeted towards the vasculature
Source: Diabetologia. 2014 May 5;57(8):1655–63. doi: 10.1007/s00125-014-3252-6 (PMC4079948; doi:10.1007/s00125-014-3252-6)
Supplement: Supplementary file 2 — (PDF 1260 kb) [file 125_2014_3252_MOESM2_ESM.pdf]

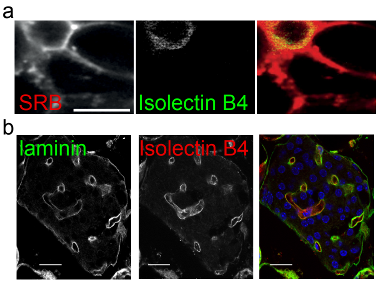


ESM Fig. 1 (a) SRB (red, extracellular dye) stained fissures also stain with isolectin B4 (green, staining) and define the residual vasculature that runs through cultured islets. (b) In fixed islet slices, laminin immunofluorescence and isolectin B4 staining outline the vasculature that runs through islets. Scale bar 10 µm.
