# Supplementary material for: Insulin secretion from beta cells in intact mouse islets is targeted towards the vasculature
Source: Diabetologia. 2014 May 5;57(8):1655–63. doi: 10.1007/s00125-014-3252-6 (PMC4079948; doi:10.1007/s00125-014-3252-6)
Supplement: Supplementary file 3 — (PDF 108 kb) [file 125_2014_3252_MOESM3_ESM.pdf]

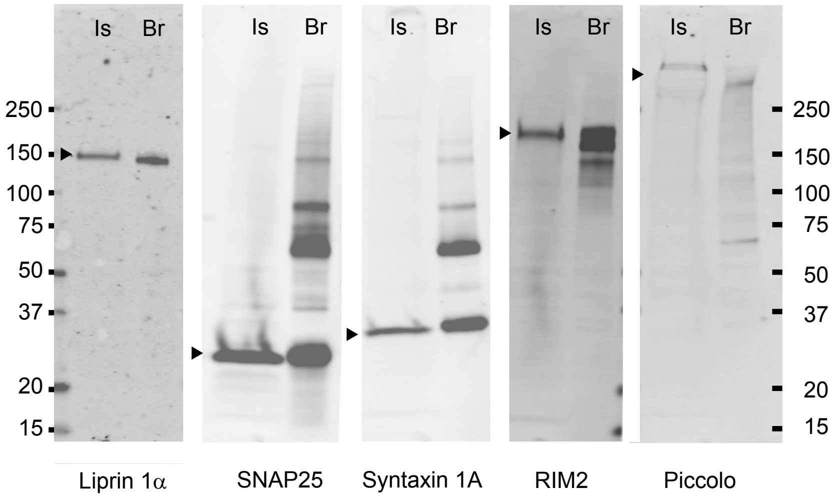

ESM Fig. 2 Samples of mouse islet protein (Is) and brain (Br) were run on polyacrylamide gels and probed using antibodies to Liprin 1α, Rim2, Syntaxin 1, SNAP25, and Piccolo. Arrowheads indicate bands of the expected molecular weights. In all cases in islets the antibodies recognised only protein bands of the expected size. Molecular weights X10-3 .
