# Supplementary material for: Insulin secretion from beta cells in intact mouse islets is targeted towards the vasculature
Source: Diabetologia. 2014 May 5;57(8):1655–63. doi: 10.1007/s00125-014-3252-6 (PMC4079948; doi:10.1007/s00125-014-3252-6)
Supplement: Supplementary file 4 — (PDF 19 kb) [file 125_2014_3252_MOESM4_ESM.pdf]

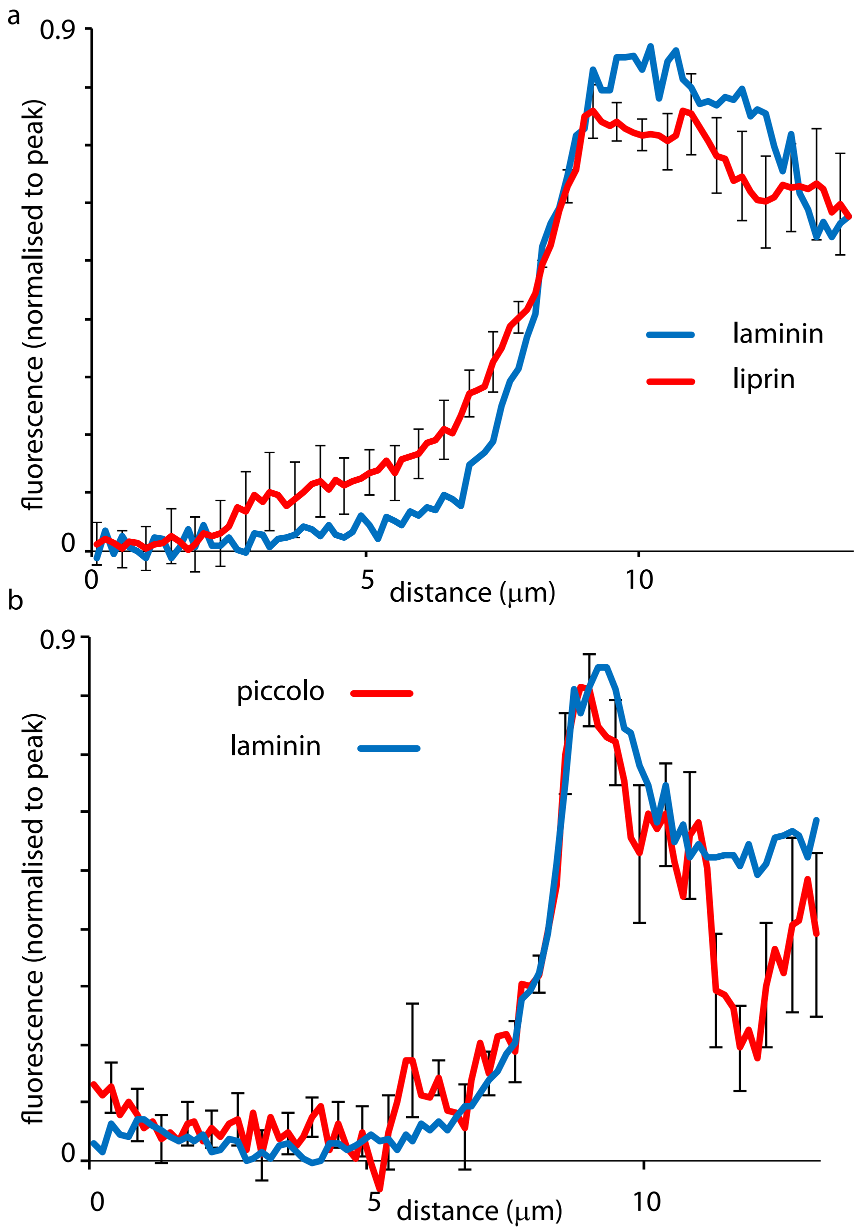

ESM Fig. 3 Linescan analysis of (a) liprin with laminin and (b) piccolo with laminin. The average fluorescence along a line drawn around the perimeter of beta cells (n=10 cells) were aligned for the mid-point of the enhanced laminin fluorescence observed at the vascular face. The mean +/- SEM for liprin and piccolo fluorescence are shown and demonstrate they are both enriched at this vascular face.
